# The Role of Bacteria in Acute Oak Decline in South-West Poland

**DOI:** 10.3390/microorganisms12050993

**Published:** 2024-05-15

**Authors:** Miłosz Tkaczyk, Katarzyna Sikora

**Affiliations:** Forest Protection Department, Forest Research Institute, ul. Braci Leśnej 3, 05-090 Sękocin Stary, Poland

**Keywords:** *Brenneria goodwinii*, oak dieback, pathogenicity, bacteria, *Gibbsiella quercinecans*

## Abstract

The process of multifactorial oak disease has been of interest to scientists from all over the world for many years. Recently, a new phenomenon has been added to the model related to oak decline, acute oak dieback, which causes oak decline and was first described in the UK. This study presents research on this phenomenon in the area of the largest oak stand complex in Poland, the so-called Krotoszyn Plateau. This work was carried out in two stages. In the first stage, 54 trees were tested for the presence of bacteria using molecular biology (real-time PCR). Subsequently, a tissue fragment was taken for inoculation from the trees in which the presence of *B. goodwinii* and *G. quercinecans* bacteria was confirmed. The isolates obtained were used to test Koch’s postulates and for biochemical analyses for Polish strains. In addition, the results obtained were also compared with the presence of *A. biguttatus*, which is considered a bacterial vector, which, in turn, confirmed that the bacteria responsible for the AOD phenomenon can also be present in trees not inhabited by this insect. The results obtained confirmed the presence of bacteria and their potential to cause necrosis in oaks, which fits into the model of the spiral disease that has been causing mass mortality of oaks in this naturally and economically valuable area since the 1980s.

## 1. Introduction

Native oak species in Poland include English oak (*Quercus robur*) and sessile oak (*Quercus petraea*). The red oak (*Q. rubra*) was introduced into Polish forests as an admixture on poor sites [1]. Both native species (*Q. robur* and *Q. petraea*) are important both from economic (wood production) and ecological points of view [2]. The phenomenon of deciduous tree dieback should be considered a complex disease determined by the interaction of a number of predisposing, initiating, and contributing factors [3]. In oaks, disease-promoting factors include prolonged drought, fluctuations in the water table, frosts, and damage by fungi and insects from the group of primary pests. It is assumed that the problems in oak stands in Poland, especially on the Krotoszyn Plateau, began in the winter of 1978/1979; the stands were flooded after the 1980 floods, and their health was most severely affected by the prolonged lack of precipitation in 1981–1983, which led to a drop in the groundwater level [4,5,6]. The first signals about the death of deciduous trees came from all over the country in the autumn of 1983. The dying off of branches and twigs, the deformation of shoots, the thinning and discolouration of leaves, and then the thinning of the crown were observed. The next reports from 1984 showed an increase in the area of deciduous tree stands with symptoms of dieback. The widespread occurrence of forest dieback has prompted many countries to carry out systematic inventories of the health status (degree of damage) of tree stands. In Poland, the degree of damage to tree stands (including oaks) has been assessed since the late 1980s using the bioindication method based on the crown transparency. This is in line with the European standards [7].

Further deterioration in the health of oak stands and the onset of oak dieback in Poland on an unprecedented scale occurred in the mid-1980s. Tree mortality, at that time, was indeed a catastrophe and affected a total of 145,000 ha of forest stands [8], i.e., about 2% of Poland’s forest area at that time. The symptoms associated with crown damage and oak dieback are much more pronounced in older stands [9]. The multiple symptoms associated with the decline and dieback of *Q. robur* include leaf discolouration followed by wilting and defoliation, stem cracking, and associated bleeding, internal bark necrosis (lesions), often indicating stem colonisation by *Agrilus biguttatus* Fabricius and callus repair [10]. Oak dieback is thought to have a complex aetiology involving dynamic interactions between trees (e.g., competition) as well as abiotic and biotic factors [11]. Furthermore, this phenomenon is thought to be a consequence of predisposing factors, including soil properties (such as poor fertility, drainage, moisture, retention, and compaction) and the role of rhizosphere microflora [10].

Among the biotic factors causing tree mortality, pathogenic bacteria responsible for the phenomenon of Acute Oak Decline have been increasingly mentioned in recent years [12,13]. Trees affected by Acute Oak Decline show abundant discharge between bark cracks. These are initially necrotic areas directly under the bark, which develop into fluid-filled cavities that emerge from under the bark in spring [13]. Over time, the secretion dries and forms shiny, sticky droplets on the bark surface. Recent studies have identified *Gibbsiella quercinecans*, *Rahnella victoriana*, and *Brenneria goodwinii* as the main causes of AOD in the UK [10,14]. These bacteria can be spread by wind or rain [12], but the most common route of transmission is insects. The presence of bacteria has been repeatedly found in the galleries of the larvae of *Agrilus biguttatus* [10,13].

The aim of this work is to understand the extent of the phenomenon and the potential threat posed by the bacteria responsible for acute oak dieback on the Krotoszyn Plateau. This will be verified through tests on weakened trees showing symptoms of acute oak dieback and pathogenicity tests to confirm whether the bacteria present in Poland can pose a threat to native oak species.

## 2. Materials and Methods

### 2.1. Symptoms and Plant Sampling

After consultations with forest managers, the decision was made to select four mature English oak stands in the south-western part of Poland, on the so-called Krotoszyn Plateau, where a deterioration in the health of the trees has been observed over the last ten years. All selected stands were older than 100 years (142 years for the first plot, 138 years for the second plot, 163 years for the third plot, and 153 years for the fourth plot). Only English oak grew in all the selected stands. Although the stands were characterised by a weakened condition and contained symptomatic trees (e.g., yellowing of leaves, crown transparency, and aerial cankers), the dieback symptoms were not observed uniformly in all stands; apparently, healthy individual trees without visible crown symptoms occurred everywhere.

The samples for the tests were taken in July 2023. The work was carried out over two steps. First, swabs were taken with sterile swabs from all trees with characteristic symptoms. The swabs were then processed according to the protocol described by [15]. Real-time PCR analysis was then performed and used to select trees for the second phase of work. In the second phase, samples were taken from trees, wherein the presence of bacteria responsible for the AOD phenomenon was confirmed (using the real-time PCR technique), to isolate pure cultures. The diseased parts were removed from the trees using sterile tools (the samples of 54 symptomatic trees were taken from 4 different plots), packed in paper bags, and stored at 4 °C for further analyses. The complete characteristics of the trees and their symptoms are shown in Table A1 in the Appendix A.

### 2.2. STAGE I—Real-Time PCR

Real-time PCR assays were performed using a Rotor-Gene^®^ thermal cycler (QIAGEN, Hilden, Germany), while the end-point PCR was performed using a Veriti™ 96-well Fast Thermal Cycler (Thermo Fisher Scientific, Waltham, MA, USA). For the *B. goodwinii* assay, the primer pair Bg99F/Bg179R and the hydrolysis probe Bg124P were used [15]. The real-time PCR reactions were performed with 19 μL of master mix consisting of 0.6 μM of forward primer, 0.6 μM of reverse primer, 0.3 μM of probe, and 1× PCR buffer (LuminoCt^®^ qPCR ReadyMix™, Sigma Aldrich, St. Louis, MO, USA), molecular-grade water, and 1 μL of bacterial cell suspension. The conditions for real-time PCR were as follows: 95 °C for 3 min, followed by 40 cycles of 95 °C for 15 s and 63 °C for 40 s. Fluorescence measurement was performed at the end of each cycle. For the *G. quercinecans* assay, the primer pair GQ_gyrB_qPCR_F/GQ_gyrB_qPCR was used [16]. The real-time PCR reactions were performed with 18 μL of master mix (consisting of 0.15 μM of forward primer, 0.15 μM of reverse primer, and 1× PCR master mix (LuminoCt^®^ SYBR^®^ Green qPCR ReadyMix, Sigma Aldrich), molecular-grade water, and 2 μL of bacterial cell suspension. The conditions for real-time PCR were as follows: 95 °C for 5 min, followed by 40 cycles (95 °C for 15 s and 62 °C for 30 s). Fluorescence was measured at the end of each cycle. Melting analysis was performed immediately after PCR in the same reaction vessel. In this procedure, the amplicon DNA was heated stepwise from 50 °C to 95 °C. The PCR products were analysed after electrophoresis through a 1% agarose gel.

To confirm the results of the screening PCR tests, both strands of the PCR products at Genomed (Warsaw, Poland) were sequenced using a 3730XL DNA analyser (Applied Biosystems, Foster City, CA, USA). The nucleotide sequences were read and manually edited with FinchTV v. 1.4.0 (Geospiza Inc., Seattle, WA, USA) and aligned with the sequences available in the GenBank database (http://www.ncbi.nlm.nih.gov (accessed on 12 May 2024)) using the BLASTn algorithm to confirm the taxonomy of the analysed bacteria based on 99% sequence similarity.

### 2.3. STAGE II—Bacterial Isolation

At sites where characteristic exudates were observed, the bark was exposed and small sections were taken with a sterilised knife from the area where the necrotic tissue met the healthy bark and sapwood tissue. The removed samples were packed in sterile plastic bags and stored in the refrigerator until the samples were transported to the laboratory. In the laboratory, the collected samples were washed, surface-sterilised in 1% sodium hypochlorite for 1 min, and rinsed with sterile distilled water [17]. From each sample collected in the field, two smaller fragments were cut with a sterile scalpel and then macerated in 2 mL of distilled water for 30 min. A loop of the resulting suspension was inoculated onto a medium containing semi-selective agar with eosin methylene blue (EMB—an isolation medium for rapid identification and differentiation of *Enterobacteriaceae*). The cultures were incubated at 28 °C for 24–48 h. After this time, the colonies formed were isolated and purified from each plate. The isolates were transplanted onto NA medium and stored at 28 °C for further analyses, while duplicates were stored at 4 °C for long-term use.

### 2.4. Biochemical Characterisation of Bacteria

The biochemical tests were carried out according to [18]. After performing the Gram stain test, the biochemical and physiological kit API 20E (bioMérieux, Lyon, France) was used for selected isolates. This test includes the following biochemical determinations: enzyme activity: ß-galactosidase, arginine dihydrolase, lysine decarboxylase, ornithine decarboxylase, urease, tryptophan deaminase, gelatinase, cytochrome oxidase, citrate utilisation, production of $H_{2}S$, indole, and acetoin (Voges Proskauer); fermentation of polysaccharides: glucose, mannitol, inositol, sorbitol, rhamnose, sucrose, melibiose, amygdalin, and arabinose. Based on these analyses, groups of isolates were identified, and then, a representative was selected to perform molecular analyses.

### 2.5. Molecular Characterisation with 16S rRNA Gene Sequencing

For bacteria isolated from oak tissue, an aliquot of 20 μL of each strain-pure bacterial preculture on NA agar media was transferred to a 2 mL Eppendorf tube containing 1 mL of NA media and incubated at 28 °C for 48 h at 125 rpm. The cultures were centrifuged at 11,500× *g* for 5 min, the supernatant (SN) was discarded, and the resulting pellet was used for further DNA extraction steps using the GenElute Bacterial Genomic DNA Kit (Sigma Aldrich) according to the manufacturer’s instructions. The extracted DNA was quantified using a NanoDrop ND-1000 droplet spectrophotometer (Thermo Scientific™, Waltham, MA, USA). All DNA was stored at −20 °C prior to analysis.

The amplification of the 16S rDNA was performed in an end-point PCR with the universal primers 27 F (5′-AGAGTTTGATCCTGGCTCAG-3′) and 1492 R (5′-TACGGYTACCT TGTTACGACTT-3′), which cover almost the entire length of the 16 S rRNA gene [19], with an expected amplicon of 1400 bp. The primer 27 F/1492 R is the most commonly used primer for species-level identification [20]. Each 25 μL of PCR reaction mixture contained 2.5 μL of 10× Taq buffer, 1 μL of 25 mM ${\mathrm{MgCl}}_{2}$, 0.5 μL of 10 mM dNTPs, 1 U Taq polymerase (QIAGEN), 0.4 μM of each primer, and 10 ng of DNA template brought to a final volume of 25 μL with ultrapure water. Reactions were performed on a Veriti™ 96-well thermal cycler (Applied Biosystems) with the following cycling conditions: initial denaturation at 94 °C for 5 min, followed by 40 cycles of denaturation at 94 °C for 30 s, annealing at 55 °C for 1 min, and extension at 72 °C for 1 min. A final extension was performed at 72 °C for 10 min. Aliquots of 2 μL of each reaction were analysed on a 1% (*w*/*v*) agarose gel in TBE buffer.

PCR amplicons of 16 S rDNA were sequenced using a 3730 XL DNA analyser (Applied Biosystems) at Genomed (Warsaw, Poland). Nucleotide sequences were read and edited using FinchTV v. 1.4.0 (Geospiza Inc.) and aligned to the reference sequence database in GenBank (http://www.ncbi.nlm.nih.gov (accessed on 12 May 2024)). The similarity search with the entries in the database was carried out using the online search BLASTn. The sequences obtained were also aligned with the 16S rDNA sequences of the type species (https://lpsn.dsmz.de/genus (accessed on 12 May 2024)).

### 2.6. Pathogenicity Test

To confirm pathogenicity and identify bacterial strains, pathogenicity tests were carried out on three-year-old English oak seedlings grown from seed under greenhouse conditions. The bacterial suspension used for inoculation was prepared from a 48 h culture (broth medium, 28 °C) adjusted to 1 × $10^{7}$ CFU/ml in sterile distilled water [18]. A total of 100 μL of the prepared suspension was taken and injected into a centimetre-long incision made with a sterile scalpel on the stem of oak seedlings kept in a greenhouse. Sterile distilled water was used to inoculate the control seedlings. Ultimately, an experiment with four groups was adopted: a control, two test experimental variants corresponding to two different bacterial strains isolated from infected oaks, and a test experimental variant in which the seedlings were inoculated simultaneously with both bacterial strains. For each group, 10 seedlings were inoculated. The injection site of the suspension was protected with parafilm, and then, all seedlings were kept at a temperature of 20–25 °C for 1 month, and the development of symptoms was recorded daily. Reisolations performed on SNA medium from symptomatic seedlings consistently yielded typical cream-coloured bacterial colonies identical to the colonies used for inoculation and consistent with Koch’s postulates.

In addition, the severity of the infection was assessed throughout the duration of the experiment (three months). Observations were carried out once a week, starting seven days after inoculation. The severity was assessed using the following scale: 0—symptomless plants, 1—leaf discolouration, 2—wilting or dying, and 3—dead plants [21]. For each assessment, the plants in each class were counted, and then, the average value for each variant was calculated. In this way, it was possible to calculate the area under the disease progression curve (AUDPC) [22]. The area under the disease progression curve (AUDPC) is a useful quantitative summary of disease intensity over time that allows for comparisons of the rate of disease progression. The most commonly used method for estimating AUDPC, the trapezoidal method, involves discretising the time variable (in the present experiment—days) and calculating the average disease intensity between each pair of adjacent time points [23]. All necessary calculations were performed using the Agricolae package for R, which includes the AUDPC function.

## 3. Results

### 3.1. Identification of Obtained Isolates

**Figure 1:**
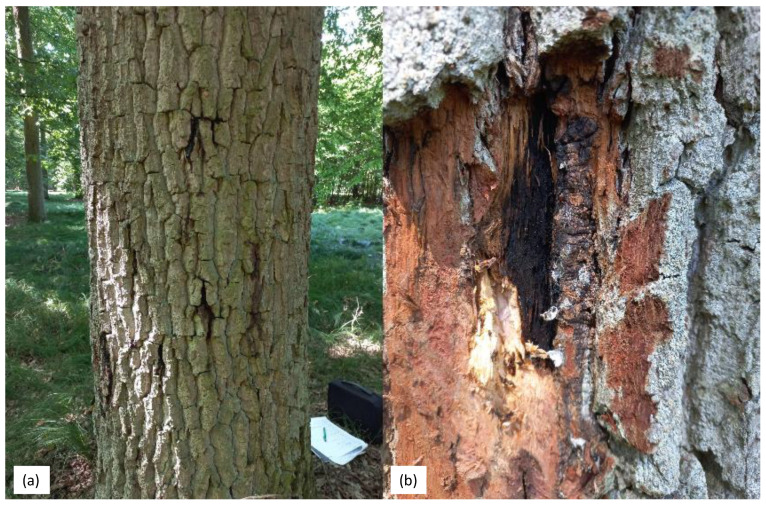
(**a**,**b**) Longitudinal cracks on the trunks of common oak (*Q. robur*) trees showing brown to black lesions accompanied by exudation.

Typical symptoms of bacterial canker were observed on English oaks in certain areas of the Krotoszyn Plateau. These symptoms included weak trees and brown to black lesions on the boot, accompanied by the release of black sap in spring, summer and autumn. The affected trees showed a progressive loss of vigour and a decline in foliage. In some cases, the bark cankers were clearly visible and reached up to 10 cm (Figure 1a,b). Of the 54 trees selected for analysis, the presence of *B. goodwinii* was confirmed in 24 trees. *G. quercinecans* was confirmed on 12 trees. The data that refer to the results of the real-time PCR method are presented in Table A1 in Appendix A. In the second work phase, in which tissue was taken from trees where the presence of the bacteria mentioned was confirmed, isolates were only obtained for *B. goodwinii* and *G. quercinecans*, and these isolates were subjected to further analyses. A total of 12 isolates described as *B. goodwinii* and 6 described as *G. quercinecans* were obtained. Both bacteria together were confirmed only in four trees. These trees had varying numbers, but importantly, of all the trees from which isolates were obtained, only two showed signs of *Agrilus biguttatus* feeding.

The nucleotide sequences were read and manually edited using FinchTV v. 1.4.0 and aligned with the sequences available in the GenBank database (http://www.ncbi.nlm.nih.gov (accessed on 12 May 2024)) using the BLASTn algorithm to confirm the taxonomy of the bacteria analysed based on 99% sequence similarity. The PCR reaction using the universal primers 27 F and 1492 R yielded a DNA fragment corresponding to a 16 S rRNA barcode region approximately 1396 base pairs in length. This specific region serves as a molecular marker for bacterial taxonomic identification. The consensus sequence obtained from the PCR product for *B.goodwinii* (GenBank accession number: PP000493) was 1395 nt long and 99.93% identical to the *B.goodwinii* sequences (KM032271) in the NCBI database. The consensus sequence obtained from the PCR product for *G.quercinecans* (GenBank accession number: OR999889- 91) was 1396 nt long and 99.93% identical to the sequences of *G.quercinecans* (GenBank accession number: MN822731).

### 3.2. Biochemical Characteristics

After carrying out biochemical and physiological tests, two types of Gram-negative bacteria were isolated, which differed slightly in appearance. Biochemically, the two bacteria were very similar; using API 20 E, they were found to be positive for beta-galactosidase and mannitol, sorbitol, inositol, rhamnose, sucrose, melibiose, amygdalin, and arabinose, and negative for oxidase, arginine dihydrolase, lysine decarboxylase, ornithine decarboxylase, $H_{2}S$ production, urease, tryptophan deaminase, indole, and gelatinase, differing only with respect to citrate. Based on their phenotypic characteristics, the bacterial isolates from oaks formed a single group that clearly shared biochemical and phenotypic characteristics with other members of the Pectobacteriaceae, particularly *Brenneria goodwinii* and *Gibbsiella quercinecans* (Table 1).

### 3.3. Confirmation of Pathogenicity

**Figure 2:**
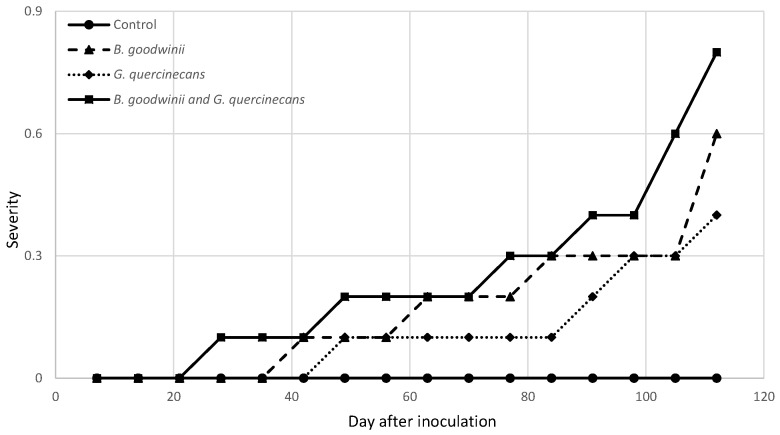
Disease severity of bacteria isolates 120 days after inoculation. Severity was evaluated using the following scale: 0—asymptomatic plants; 1—leaf discolouration; 2—wilting, dieback; and 3—dead plant.

The severity of the infection and the calculated AUDPC values are shown in Figure 2. In the variant in which the plants were inoculated simultaneously with *B. goodwinii* and *G. quercinecans*, the first symptoms of infection were observed after four weeks (leaf discolouration). The AUDPC value for this variant was the highest at 24.5 (on the day the trial ended). In the variant in which the oaks were inoculated with *B. goodwinii*, the first symptoms were observed on day 42 of the experiment and, in the case of *G. quercinecans*, 1 week later (day 49). At the end of the experiment, the AUDPC values for *B. goodwinii* and *G. quercinecans* were 16.8 and 11.2, respectively. It is worth mentioning that during the four months of the experiment, no seedling mortality was observed in any of the tested variants.

**Figure 3:**
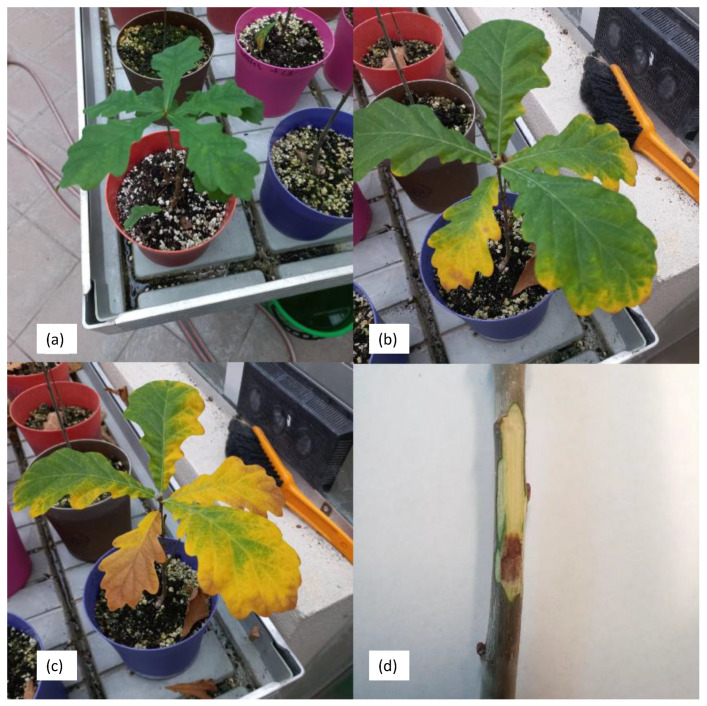
Change in the colour of the leaves of an oak seedling with the variants *B. goodwinii* and *G. quercinecans*; (**a**) on the day of inoculation, (**b**) five weeks after inoculation, (**c**) on the day of termination of the experiment; (**d**) canker symptoms after one month of artificial inoculation on the trunk of an oak seedling.

Four months after the inoculation of the seedlings, small necrotic lesions appeared on the bark around the inoculation sites and brown necrotic spots in the inner part of the wood (Figure 3). The bacteria were again isolated from the diseased tissues and identified as *B. goodwinii* and *G. quercinecans* based on colony morphology, biochemical analyses and PCR amplification. The control seedlings treated with SDW showed no symptoms.

## 4. Discussion

Very large complexes of dense oak forests in Poland, with an area of over 4000 m^2^. ha, which are over 100 years old, occur in the so-called Krotoszyn Plateau [25]. Thanks to this, the Krotoszyn forests have long aroused the interest of naturalists and foresters [26] and are considered one of the most valuable oak forest refuges in Central Europe. The preservation of these forests in the midst of intensively farmed fields is unique [27]. The Krotoszyn oaks are usually identified with the areas a short distance south and north-east of Krotoszyn, mainly because the oak stands there occupy the largest area and are the most characteristic element of the forest environment. Due to its uniqueness and undeniable natural and economic values, the Krotoszyn Oak Forest has been and still is the object of pride and constant care of foresters. The oak stands on the Krotoszyn Plateau were repeatedly affected by a number of biotic and abiotic factors that caused the so-called oak dieback. Many of these negative phenomena were global and affected the whole of Europe or a large part of it.

For many years, the frequent occurrence of fungi of the genus *Armillaria* spp. has been observed in damaged tree stands, which become significantly more active when the trees are physiologically weakened due to drought. As a natural component of forest habitats, these fungi develop saprotrophically, i.e., they remain in the ecological balance of the pathogen–host–environment relationship, but when this balance is disturbed, they enter the parasitic phase [6]. Physiological disturbances increase the susceptibility of the host to disease, which is immediately exploited by fungi of the genus *Ceratocystis* and *Ophiostoma*, which cause vascular diseases [28,29] and damage the assimilation apparatus (*Erysiphe alphitoides*), and then, fungi cause internal decay, which leads to the degradation of wood [30]. In the process of tree death, the role of insects causing defoliation (*Tortricidae* and *Geometridae*) and secondary pests (*Agrilus*) should not be overlooked [31]. The interaction of all damaging factors ultimately leads to the weakening and separation of the trees in the stand [32]. In recent years, the above-mentioned list of pests responsible for the decline in oaks on the Krotoszyn Plateau has been joined by a number of pathogens of the genus *Phytophthora*, which are responsible for damage to the root systems [33].

This paper presents studies aimed at confirming the presence of *B. goodwinii* and *G. quercinecans* bacteria in exudates from dying oak trees on the Krotoszyn Plateau. In contrast to previous studies [34], the analysis for the presence of bacteria was carried out on a larger number of trees, namely, in massive oak stands over 100 years old. The analyses carried out allowed the isolation of *B. goodwinii* and *G. quercinecans* bacteria, which made it possible to perform pathogenicity tests to verify Koch’s postulates. This research will help to raise awareness of the presence of these bacteria in Poland as well. The current analyses cover a larger area, allowing isolates to be obtained from infected tissue, which enabled an in-depth analysis of the occurrence of the AOD phenomenon in oak stands in Poland.

Acute Oak Decline was first observed in native oaks in the UK. The main symptoms are bleeding bark cracks associated with insect galleries, crown thinning, and epicormic shoots, and death can occur within 4–5 years [10]. Since then, the presence of these pathogenic bacteria has been confirmed in many other countries, including Poland, Latvia, Portugal, Spain, and Switzerland [34,35,36,37]. Four bacteria are commonly recovered from bleeding cracks: *G. quercinecans*, *B. goodwinii*, *R. victoriana*, and *L. britannica*. Although all four are associated with AOD, the most commonly encountered bacteria are *G. quercinecans*, *B. goodwinii*, and *R. victoriana*, with *G. quercinecans* clearly present, and especially *B. goodwinii* [38], 2018), which has been shown to have the ability to cause wood necrosis [39]. Of these bacteria, *B. goodwinii* has been identified as a major component of the erosion microbiome, both through pathogenicity tests on strains [39] and gene database comparisons that revealed its repertoire of virulence genes that give it the ability to persist on oak stem tissue [40]. Meanwhile, *G. quercinecans* and *R. victoriana* appear to be secondary components of the erosion microbiome, increasing the severity of erosion through different genes, but are not essential for erosion formation [39]. The results of the research described above are largely consistent with the results of the experiment presented. The AUDPC analysis showed that the seedlings that suffered the most belonged to the variant in which both bacteria (*B. goodwinii* and *G. quercinecans*) occurred together. Oak seedlings infected simultaneously with both bacteria showed changes in the assimilation apparatus as early as 4 weeks after the bacteria were introduced into the strains. When the bacteria occurred individually, *B. goodwinii* was much more pathogenic. As for *G. quercinecans*, disease symptoms were also observed in this variant after 42 days, but only in 3 out of 10 seedlings by the end of the experiment. Previous studies have confirmed that the development of AOD has a polymicrobial cause, and multiomics data have confirmed that *B. goodwinii* is an important bacterial pathogen in this process [38,41]. Bacteria of the genus *Brenneria* are widely known as parogenes of various woody plants, including willow—*B. salicis*, lime—*B. tiliae*, walnut—*B. nigrifluence*, poplar—*B. populi*, and alder—*B. alni* [42,43,44,45]. This is why reports of further pathogenicity tests for *B. goodwinii* seem so important. In the present work, this bacterium, which is difficult to cultivate outside of tissues, was successfully isolated, making it possible to test the virulence of this particular Polish strain.

The symptoms associated with the presence of bacteria in dying oak stands in western Poland are consistent with those described in the above papers. This may indicate that in the multifactorial process of oak stand decline in this area, in addition to the factors mentioned above, new factors, namely, the bacteria *B. goodwinii* and *G. quercinecans*, should also be taken into account. This hypothesis is confirmed by the pathogenicity results presented in this article, which indicate that they can cause necrosis and weakening of entire plants. The confirmation of the pathogenicity of these bacteria under Polish conditions and the association of their occurrence with the AOD phenomenon fits into the new model of spiral disease described by Denamn et al. [46]. In their study, the authors updated the spiral disease model, which emphasises the predisposition of trees to disease through changes in soil, climate, pollutants, primary root pathogens, management, tree age and genetic potential. The predisposition combined with triggering factors such as repeated leaf infestations, disturbances, prolonged droughts or floods, and then contributing factors, namely, biotic pathogens, lead the tree from a healthy state to a state of severe degradation and eventually death [46]. In this model, the bacterial complex discussed in this study was mentioned as one of the biotic components involved in oak stem erosion.

Another important factor in the research described is the joint interaction of factors related to the dying process, the so-called combination of insects and bacteria. There is still debate in the literature about the possible sources of bacterial spread. *Agrilus biguttatus* is one example [47], although there is no clear empirical evidence of its role as a vector of necrotrophic bacteria [13]. It is hypothesised that beetle larvae transport AOD-related bacteria through the inner bark when forming larval galleries, as these are often located close to necrotic lesions, and bacteria are isolated along the galleries [24]. However, this is not evident from the studies presented. The presence of the mentioned *A. biguttatus* galleries was only confirmed on a few trees. The bacteria were observed much more frequently in trees where there were no signs of insect feeding, which could indicate that, despite various reports, there is no correlation between the presence of bacteria and the colonisation of trees by *A. biguttatus* under the conditions observed on the Krotoszyn Plateau.

This fact can be explained based on the research of Brober et al. [38], in which it was found that *B. goodwinii* is a specific wood endosymbiont that, when the tree is weakened and conditions are favourable, can become a pathogen leading to the death of the host plant. Furthermore, the results of these studies explain why this bacterium is rapidly decomposed outside the oak host in rainwater and forest soil. The results obtained in this study confirm the thesis put forward by Brober et al. [38]. The identified bacteria occurred both in trees where insect activity was observed, in trees with many exudates, and in trees where no exit holes were observed, and the exudates were taken from old, inactive exudates. This could indicate that the exudates were caused by other factors such as *Armillaria*, *Phytophthora*, or other abiotic factors [10]. The bacteria confirmed in these trees were present in the form of endophytes that had not switched to pathogenic due to the lack of optimal conditions.

Our study confirmed that *B. goodwinii* and *G. quercinecans* bacteria might represent another biotic factor that fits into the multifactorial disease model. However, this study also shows that further work is needed on the population dynamics of these bacteria and their interactions with other organisms in the environment. Understanding the ecology and biology of these pathogens could help to develop more effective methods to combat them and protect the vegetation on the Krotoszyn Plateau. It would also be valuable to carry out genetic analyses of isolates of these bacteria to better understand their origin and possible relationships with other bacterial populations. This can provide valuable information on transmission routes and potential sources of contamination, which can ultimately lead to more effective preventive measures.

## Figures and Tables

**Table 1:** Biochemical characteristics of the Polish oak isolates and reference strains of *Brenneria goodwinii* and *Gibbsiella quercinecans* species using the API 20 E. (marked with a “+” sign in case of a positive result, “-” negative result).

Test	*Gibbsiella quercinecans* (OR999889)	*Gibbsiella quercinecans* (OR999890)	*Gibbsiella quercinecans* (OR999891)	*Brenneria goodwinii* (PP000493)	*Gibbsiella quercinecans*	*Brenneria goodwinii*
β-galactosidase	-	-	-	+	-	+
Arginine dihydrolase	-	-	-	-	-	-
Lysine decarboxylase	-	-	-	-	-	-
Ornithine decarboxylase	-	-	-	-	-	-
Citrate utilisation	-	-	-	-	-	-
$H_{2}S$ production	-	-	-	-	-	-
Urease	-	-	-	-	-	-
Tryptophane deaminase	-	-	-	-	-	-
Indole production	-	-	-	-	-	-
Acetoin production	-	-	-	+	-	+
Gelatinase	-	-	-	-	-	-
Assimilation of:						
Glucose	-	+	+	+	+	+
Mannitol	+	+	+	+	+	+
Inositol	+	+	+	+	+	+
Sorbitol	+	+	+	+	+	+
Rhamnose	+	+	+	+	+	+
Sucrose	+	+	+	+	+	+
Melibiose	+	+	+	-	+	+
Amygdalin	+	+	+	+	+	+
Arabinose	+	+	+	+	+	+
Oxidase	-	-	-	-	-	-
Gram reaction	-	-	-	-	-	-
Reference					[24]	[24]

## Data Availability

Data are contained within the article.

## Appendix A. Results of Real-Time PCR

**Table A1:** Real-time PCR result (marked with a “+” sign in case of a positive result), characteristics, and conditions of trees.

DBH (cm)	Estimated Height (m)	Additional Info.	*Brenneria goodwinii*	*Gibbsiella quercinecans*
Geographic coordinates of the first research area—51.723760, 17.557601				
54	26	Few bleeds (<10), exit holes not noted		+
43	27	Very few *Agrilus* exit holes evident		+
43	32	Few bleeds (<10), few *Agrilus* exit holes, Fomitiporia robusta present	+	
42	29	Few bleeds (<10), exit holes not noted	+	
46	28	Moderate bleeds (10–20), few *Agrilus* exit holes		
55	32	Moderate fresh bleeds (10–20), exit holes not noted		
41	30	Few bleeds (<10), exit holes not noted		
43	27	Few bleeds (<10), exit holes not noted	+	+
56	26	Few bleeds (<10), exit holes not noted		
54	27	Few bleeds (<10), exit holes not noted	+	
40	30	Few bleeds (<10), exit holes not noted	+	
54	29	Fresh multiple bleeds (>20), exit holes not noted	+	+
49	27	No active bleeds, exit holes not noted	+	
47	31	No active bleeds, exit holes not noted		
44	27	Moderate bleeds (10–20), exit holes not noted		+
42	30	Few bleeds (<10), very few *Agrilus* exit holes evident		
45	31	Few no active bleeds, exit holes not noted	+	
53	26	Few bleeds (<10), exit holes not noted	+	
52	26	Fresh multiple bleeds (>20), exit holes not noted	+	+
50	32	Fresh multiple bleeds (>20), exit holes not noted		+
Geographic coordinates of the second research area—51.722848, 17.548323				
47	31	Few bleeds (<10), very few *Agrilus* exit holes evident	+	
51	27	Few bleeds (<10), exit holes not noted	+	+
44	28	Very few *Agrilus* exit holes evident	+	
42	31	Fresh multiple bleeds (>20), exit holes not noted		+
39	27	Very few *Agrilus* exit holes evident	+	
54	32	Fresh moderate bleeds (10–20), exit holes not noted		
45	29	Few bleeds (<10), exit holes not noted		
39	27	Few bleeds (<10), *Fomitiporia robustapresen*, exit holes not noted	+	
44	29	Fresh moderate bleeds (10–20), exit holes not noted	+	
50	28	Few bleeds (<10), exit holes not noted	+	+
42	29	Few bleeds (<10), exit holes not noted	+	
50	28	Fresh multiple bleeds (>20), exit holes not noted		
44	32	Few bleeds (<10), exit holes not noted		
42	29	No active bleeds, few *Agrilus* exit holes evident		
52	32	Very few *Agrilus* exit holes evident		
51	29	Fresh moderate bleeds (10–20), exit holes not noted	+	
43	28	Few bleeds (<10), very few *Agrilus* exit holes evident	+	
49	31	Few bleeds (<10), exit holes not noted	+	
42	27	Few bleeds (<10), exit holes not noted	+	
50	32	Few bleeds (<10), exit holes not noted		
Geographic coordinates of the third research area—51.777169, 17.615834				
55	29	Moderate fresh bleeds (10–20), exit holes not noted	+	+
53	26	No active bleeds, exit holes not noted		
47	28	Moderate fresh bleeds (10–20), exit holes not noted		
56	25	Few bleeds (<10), very few *Agrilus* exit holes evident		
58	27	Moderate fresh bleeds (10–20), exit holes not noted		
54	26	Few bleeds (<10), exit holes not noted		
56	25	Few bleeds (<10), exit holes not noted		
Geographic coordinates of the fourth research area—51.779015, 17.578788				
48	28	Few bleeds (<10), very few *Agrilus* exit holes evident		
38	30	No active bleeds, exit holes not noted	+	+
42	26	No active bleeds, exit holes not noted		
47	27	No active bleeds, exit holes not noted		
39	24	Few bleeds (<10), exit holes not noted		
41	27	Fresh moderate bleeds (10–20), few *Agrilus* exit holes evident		
38	28	Few bleeds (<10), exit holes not noted		
